# Occupational Syndemics in Farmworkers in the Cape Winelands, South Africa

**DOI:** 10.3390/tropicalmed10070179

**Published:** 2025-06-24

**Authors:** Nicola Bulled

**Affiliations:** Institute for Collaboration on Health, Intervention, and Policy (InCHIP), University of Connecticut, 2006 Hillside Road, Storrs, CT 06269, USA; nicola.bulled@uconn.edu

**Keywords:** occupational health, HIV, tuberculosis, syndemics, wine farms, South Africa

## Abstract

Occupational exposures in the agricultural industry globally have been associated with heightened risk for several diseases. Reports written in South Africa in the last decade have raised awareness of the harsh occupational conditions and human rights abuses suffered by farmworker communities in the wine industry. Despite receiving “fair trade” labels upon reentry into the global market in the 1990s, the working conditions on wine farms in South Africa have remained unchanged and exploitative for centuries. Farmworkers remain dependent on substandard farm housing, have insecure land tenure rights, are exposed to toxic pesticides, are denied access to benefits and unionization, and endure long working hours in harsh environmental conditions with low pay. These occupational conditions are linked to interacting disease clusters: metabolic syndrome, problematic drinking, and communicable diseases including tuberculosis, HIV, and COVID-19. This milieu of interacting diseases with deleterious outcomes is an under-considered occupational syndemic that will likely worsen given both the lasting impacts of COVID-19 and more recent shifts in global public health funding.

## 1. Introduction

Agriculture is the single largest form of employment in the world, playing a role in the economies of most countries and in the lives of local populations [1,2]. Agriculture is also a hazardous occupation, with one of the highest occupational fatality, non-fatal injury, and occupational illness rates [3,4]. Over 25 years ago, a review of the links between agriculture and health by Lipton & De Kadt [5] highlighted the direct link between agriculture, disease, death via malnutrition, and chronic and infectious diseases.

Farming as a means of employment entails unique characteristics including settings, organizational structure, policies, and oversight. Farmworkers are exposed to several health hazards as a result of their professional activities, including ultraviolet radiation; inhalation of organic dust from spores and minerals; exposure to microorganisms such as viruses, bacteria, and infectious parasites, and their toxic products; and exposure to pesticides [2,4,6,7,8]. Hazards extend beyond the workplace to include limited protections in housing [9], lack of access to clean water and sanitation facilities [10,11], and inaccessible healthcare.

A number of studies have suggested a unique pattern of disease among farmers when compared to other populations [3]. A study by Davila et al. [12] established that farmworkers in the US have an increased prevalence of metabolic syndrome. Studies from around the world have documented significantly higher rates of depression, anxiety, and suicide among farmers than the general population [13,14,15,16,17,18,19]. The prevalence of chronic kidney disease of unknown etiology has been steadily increasing in agricultural communities globally [20]. Occupational and environmental exposures in agricultural settings are also known to elicit lung inflammatory responses and increase risk for numerous lung diseases including asthma, COPD, and COVID-19 [21,22].

In this paper, I present a syndemic examination of farmworkers in South Africa’s Cape Winelands, arguing that the confluence of social and biological forces that support a milieu of interacting chronic, infectious, and mental health conditions is an occupational syndemic. First, I present how the syndemic framework offers a way to comprehensively consider the increased health risks presented by occupational settings. Second, I consider historic and current labor practices and their impact on comorbid disease clusters in the Cape Winelands. Third, I argue how these labor practices support an occupational syndemic consisting of a clustering of interacting diseases (tuberculosis [TB], HIV, metabolic syndrome [MetS], problematic drinking) in socially, economically, and politically marginalized farmworkers. In so doing, I use a syndemic lens to draw attention to the complex occupational health crisis endured by vulnerable workers globally and the need for comprehensive global health and occupational reforms. Data for this paper are drawn from a variety of sources including published scientific literature, country, industry, and global health organization reports, and grey and periodical literature.

## 2. Applying the Syndemics Framework to Understand Occupational Risks

Syndemics theory has gained traction in medical and public health applications, with a tenfold rise in related published scientific output in the past decade [23,24]. It offers a comprehensive and contextualized understanding of adverse disease clusters in marginalized populations [25]. The concept has informed policy and programming on numerous disease epidemics including most recently MPOX [26], COVID-19 [27], and Ebola [28]. Understanding the biological and social elements of a syndemic can inform a comprehensive response to address individual disease elements, relationships between disease conditions, and the social determinants of health [29,30,31].

Syndemics theory provides a critical alternative to the concepts of comorbidities and social determinants of health by recognizing two detrimental elements simultaneously: (1) the driving effects of unequal and unjust social and structural determinants resulting in disease clusters, and (2) the interaction of coexisting diseases resulting in excess health burden beyond comorbidity. In this, the objective of the syndemic framework is to understand the whole health picture and the alignment of its constituent biological and social/structural elements. In the investigation of these issues, the syndemics approach addresses three primary questions: (1) How do diseases interact in ways that enhance harm? (2) How do social and structural conditions promote disease clustering and disease interaction? (3) How can we prevent and mitigate syndemics?

Scholars have recently applied the syndemic framework to address disease clusters in specific occupational settings, driven by observations of excess COVID-19 burden. For example, Lemke et al. [32,33] presented an argument for COVID-19-related syndemics in long-haul truck drivers in the US. Truck drivers experience higher rates of COVID-19 infection, morbidity, and mortality, overlapping with already disproportionate health burdens of cardiometabolic and respiratory comorbidities, including obesity, hypertension, diabetes, bronchitis, emphysema, and lung cancer. The COVID-19 syndemics in this population are shaped by the unique occupational configurations of long-haul trucking, including harmful work organization characteristics, toxic workplace environments, and structural factors. Long periods of time away from home, excessive time pressures, shift work, and non-working hours in worksite environments encourage detrimental lifestyle and behavioral patterns such as poor diet, lack of physical activity, poor sleep health, and high rates of cigarette smoking. Workplace environments expose truck drivers to noxious air pollutants such as carbon monoxide, nitrogen oxides, and particulate matter, including road dust. Compounding the health ramifications of these occupational factors is limited healthcare access, reflected in both poor insurance coverage and lack of available medical services, leading to failures in screening, diagnosis, and treatment for acute and chronic conditions.

Studies of Mexican migrant farm laborers in the US indicated a two-to-three-times-higher rate of COVID-19 infection, morbidity, and mortality among farmworkers than the general population, a population already heavily burdened by high rates of food insecurity, stress, depression, anxiety, obesity, high blood pressure, and diabetes. Singer and Cook [34] linked these disproportionate health burdens to occupational exposures, as the employment sector was deemed “essential” during the early years of the COVID-19 pandemic when preventive measures such as vaccines were not available. In addition, the nature of agricultural labor and related communal living increases airborne disease transmission through close contact and confined shared spaces. Furthermore, structural factors include the historical exclusion of farmworkers from health and labor protections, low wages, limited access to healthcare due to travel distances related to the rural nature of farmwork; the cost of healthcare, with few farm contracts including healthcare insurance; and the fear of engaging with healthcare institutions given the lack of legal documentation or ill-defined temporary work permits.

Singer [35] presented an empirical argument for the syndemic nature of comorbid disease conditions experienced by commercial fishers. He argued that the occupational characteristics of commercial fishing contribute directly to excess health burdens including substance abuse, violence, mental health disorders, injuries, and chronic pain, as well as COVID-19 infection, morbidity, and mortality. The physically demanding and dangerous working conditions of commercial fishing and crowded working sites, as well as the monotony of the work, the social isolation, sleep deprivation, fatigue, and economic uncertainty, promote detrimental behavior such as poor diets, alcohol, tobacco, and drug use as well as increase exposure to airborne disease.

The historical labor policies of the mining sector in South Africa, including short-term migratory labor contracts, low wages, crowded and unsanitary communal housing, limited family accommodations, and risky working conditions, are driving features of high levels of adversely interacting disease comorbidities of TB, HIV, silicosis, and COVID-19, argue Bulled and Singer [30]. The dangerous working conditions and limited worker protections increase risk for lung diseases such as silicosis, as well as communal diseases such as TB and COVID-19, among mine laborers. While reforms have been made in the industry to address the HIV epidemic, with HIV rates high among mine laborers, the industry has not undergone comprehensive occupational reforms, failing to meet social and labor plan targets, with workers continuing to have limited access to secure job contracts, acceptable housing, and robust healthcare.

The literature on occupational syndemics to date affirms the conclusion of Abrams [36] that “if we are to understand the history of occupational health, it must be viewed in the context of the labor-capital relationship: work-related disease is socially produced and is, therefore, preventable.” However, as noted by Merrill Singer, the developer of the syndemics framework, “Despite promising initial efforts, as yet the full potential of a syndemics framework for deconstructing the complexities of occupational health under neoliberal capitalism remains unrealized” [37]. With this paper, I aim to contribute to further evidencing the application of the syndemics framework in occupational settings in which disease burdens are compounded.

## 3. Working on Cape Wine Farms

Out of South Africa’s nine provinces, the greatest number of farmworkers live in the wealthy and fertile Western Cape. South Africa’s wine industry contributed 0.9% of the national GDP, 1.8% of national employment, and ZAR 18.85 billion in household income in 2024 [38]. Its links to other industries in its value chain generates greater multiplier effects than the average South African industry, making it particularly important to the national and individual economies. Consequently, the wine industry has a greater contribution to GDP (ZAR 1.57 vs. 1.3 million) and employment (7.51 vs. 6.58 jobs) for every ZAR 1 million in output relative to the average South African industry.

In reports published in 2003, 2008, and 2011, the South African Human Rights Commission documented clear abuses of wine farmworkers and labor law violations [39]. To varying degrees, farmworkers are subject to exploitative conditions and abusive practices, perpetrated by farm owners or managers. Wine farm work is physically demanding, involving long hours in harsh weather conditions, with workers exposed to toxic pesticides that are sprayed on crops. Inadequate safety equipment, limited to only overalls and rubber gloves, in contravention of health and safety regulations, exposes farmworkers to toxic pesticides. Farmworkers report covering their faces with their caps or with ineffective dust masks in an attempt to block the spray of chemicals as they have no access to respirators [39]. Workers who work with chemicals report having no appropriate cleaning facilities to use, consequently exposing others including family members to toxic chemicals. Workers report often having no access to hand washing facilities, toilets, or drinking water, as required by labor regulations [39]. Transportation of workers on the back of trucks, a contentious but common and legal practice in South Africa, also presents a risk. In addition to accidents involving workers being flung from the backs of trucks [40], the close proximity of workers to one another in the truck bed increases the likelihood of airborne disease transmission.

Farmworkers earn among the lowest wages in South Africa and are often denied benefits to which they are legally entitled [39]. Under Sectorial Determination 13 (SD13), passed by the Department of Labor in 2003 in an effort to protect workers, employers are required to pay sick leave, annual leave, and maternity leave [39]. SD13 also requires employers to pay at least the minimum wage, limits the amount of deductions that employers can make from wages for food and accommodation, and regulates the hours worked. However, under SD13, employers are only required to pay sick leave if workers have accrued sufficient sick leave, accruing sick leave at a ratio of 1 day of sick leave for every 26 days worked [41]. Paid leave and family responsibility leave only have to be granted to fixed-term workers if their contracts are for more than four months [41]. To limit their obligations under SD13, employers opt to employ workers on short-term contracts, and when farmworkers are ill or injured, they are often denied sick leave or are required to present a medical certificate [39,41].

Farmworkers also struggle to obtain timely or affordable healthcare given their remote location, limited transportation options, and low wages [39]. A study conducted in 2003 pointed to the impact of significant health budget cuts and the centralization of healthcare delivery, reducing mobile clinic visits to farms by 64% between 1997 and 2000 [42]. The changes resulted in public clinics operating in fixed facilities or on centrally situated farms up to 5 km away and operating only on weekdays during office hours. These findings are consistent with a more recent study conducted in Limpopo, with the majority of farmworkers reporting walking 2–5 km to the nearest public health clinic, with a third living beyond a 5 km radius [43].

Farm owners are not responsible for providing housing, although many have historically done so. Moreover, there are no specific regulations governing the conditions of on-farm housing. This gap in legislation means that housing for farmworkers often is substandard [39]. Human Rights Watch reported on Isaak, a farmworker who had lived for 10 years in a former pig stall with no electricity, water, or ability to provide adequate shelter from the elements [39]. In hostels, primarily maintained for seasonal workers, conditions are often overcrowded and unclean, with reports of as many as 17 sharing a single room [41].

Farm owners have no incentive to maintain housing in good condition, as the Extension of Security and Tenure Act (no.62 in 1997) stipulates that any farm dweller, even those whose employment has been terminated, may only be evicted from on-farm housing if the farmer obtains a court order and if suitable alternative housing is available. To avoid the ESTA’s strictures, farmers appoint workers on short fixed-term rather than permanent contracts and accommodate them in on-farm hostels or not at all [41]. Farmworkers endure this housing because moving elsewhere is often not financially feasible due to both their low wages and the transportation costs of getting to work. The skewed land-holding patterns in South Africa mean that poor people in rural areas have very few options for affordable and decent housing near remote farms. In recent years, overcrowded, unsafe, and unsanitary informal settlements have been growing outside wine-producing towns [44].

Despite these evident occupational hazards, farmworkers have limited avenues to report grievances. Most workers keep quiet, fearing that if they complain they will lose their jobs or get deported if they are foreign migrant workers [41]. A study of citrus workers in the Western Cape found that about a third of workers were represented by a worker committee to which they lodged their grievances [41]. Farmers have actively attempted to block union formation on farms [39]. In the Western Cape region, unionization of farmworkers is low at 5–8%, compared to a national average of 25% [39].

## 4. Wine Farms as Disease Hotspots

There is currently limited to no literature or data available on the prevalence of diseases among farmworkers in South Africa, and even less that detail the health conditions of farmworkers in the Cape Winelands. One study in the Cape Winelands District Municipality, formerly known as the Boland District Municipality, found a high prevalence of MetS among farmworkers [45]. MetS is a cluster of risk factors including high blood pressure, elevated fasting blood glucose, increased waist circumference, and high cholesterol, that are mostly associated with people living with obesity and diabetes. Research has also shown that the wine farmworker population is chronically malnourished, despite a large portion of their income being spent on food [42]. Another study among farmworkers in the Cape Winelands District found the incidence of TB to be exceptionally high, with a reported new smear-positive TB incidence rate of 1685 per 10,000 population [46], three times the provincial rate and almost five times the national level [47,48]. Although there is a dearth of studies on TB incidence and prevalence among farmworkers in South Africa, it has been reported that there is an increased risk of TB for agricultural workers globally when compared to workers in other occupations [49,50]. According to the National Occupational Mortality Surveillance South Africa (NOMS-SA), of the 114,706 deaths due to TB reported between 2013 and 2015, agriculture constituted the largest share of TB-related deaths at 20% [51]. The odds of dying from TB are 58% higher among agricultural laborers compared to those in other occupations with odds increasing among workers exposed to silica dust [52].

High levels of exposure to silica in the agricultural industry has been reported in South Africa [53]. As noted elsewhere [30], the lung damage resulting from silica exposure likely contributed to increased COVID-19 infectivity in certain occupations in South Africa. High levels of pesticide and other chemical exposure are common on farms, known to increase asthma and other lung conditions. The limited pesticide-protective equipment and overcrowded living and transportation conditions further supports a likely higher burden of COVID-19 among South African farmworkers, as present elsewhere [22,54,55]. It is not possible to gauge whether the area’s morbidity and mortality from COVID-19 was more or less than the national, as population statistics were based on 2011 census data and not representative of current area population and, as elsewhere, statistics were probably an undercount [41].

In 2010, farmworkers were reported to have a higher burden of HIV (35–42% vs. 18%) [56,57] relative to the general South Africa population [58]. In 2001, the HIV prevalence among pregnant women attending public health antenatal facilities in Cape Winelands Municipality District was 8.3% [59]. Farmworkers have an increased HIV risk due to several factors, most notably the link between farm work and migration [60]. Migration is described as the single greatest predictor of HIV risk and prevalence in Sub-Saharan Africa [61]. In South Africa, farmworkers who are migrants (internal and foreign) and their partners have a higher burden of HIV compared with non-migrants and their partners [62,63,64].

Finally, harmful alcohol consumption and its negative impact has been identified as particularly high among farming communities in the Western Cape [65,66,67,68]. Rates of fetal alcohol syndrome or partial fetal alcohol syndrome in the province are among the highest in the world [69,70], and alcohol-related interpersonal violence and child neglect has been highlighted as serious problems [65,69,71,72]. A study on alcohol use in wine farms in Stellenbosch/Franschoek and Vredendal in the Western Cape, demonstrated a high prevalence of both current drinking (69%) and, among current drinkers, symptoms of problem drinking (73%), with the drinking scores surpassing provincial estimates by 2 for men and 3.5 times for women [68]. Farmworkers in the Cape Winelands describe their community as “characterized by excessive drinking”, with intergenerational problematic drinking, drinking-related interpersonal violence, physical violence and injury, and sexual infidelity considered norms [73]. Rather than viewing excessive drinking as problematic, wine farmworkers view drinking as serving important functions, including as a facilitator of pleasure, recreation, stress release, and social connection [73].

## 5. Occupational Syndemic of Farmworkers in the Cape Winelands

**Figure 1:**
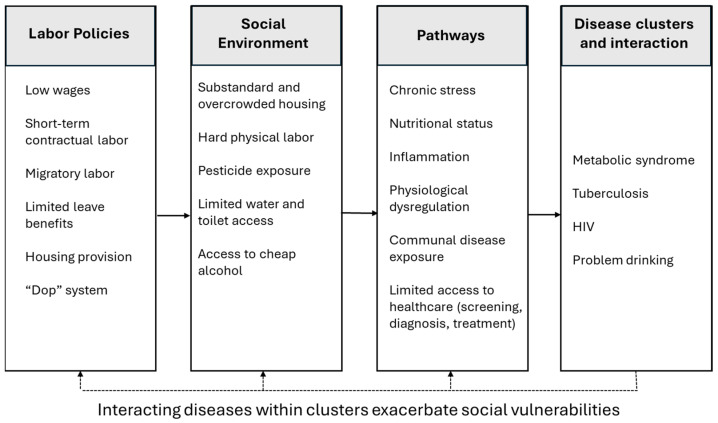
Occupational syndemic model of wine farmworkers in South Africa.

Two elements constitute a syndemic: (1) negative disease interactions within a specified population; and (2) inequitable structural relationships driving disease interaction and increased health burden. In the case of occupational syndemics, structural factors and anthropogenic environments are expressed in unsafe work and living conditions and ineffective labor policies. For South Africa’s wine industry, occupational syndemics include a high burden of MetS, TB, HIV, and problem drinking driven by high levels of exposure to pesticides damaging lung tissue, crowded and unsanitary living conditions increasing airborne disease transmission, and a historical legacy of the “dop” system supporting intergenerational excess drinking which contributes to risky sexual practices and interpersonal violence; see Figure 1. Moreover, labor policies that perpetuate low wages and substandard housing and limit workers’ ability to take sick leave, and centralized healthcare systems that limit access to health facilities, further support disease transmission and increase disease morbidity.

Addressing the first element of the syndemic—the interaction of diseases resulting in deleterious health outcomes—South African wine farmworkers experience a high prevalence of four deadly diseases (MetS, TB, HIV, problematic drinking) and these diseases are known to adversely interact. Interaction exists if the joint effect is significantly larger than the sum of the individual effects. In diseases, this can occur across multiple pathways and mechanisms, including (1) diseases/conditions that weaken the effectiveness of components of the body’s immune system; (2) diseases that damage organs and tissues, facilitating the onset of other diseases/conditions; and (3) diseases that disrupt body cellular signaling, causing a downgrading of cell functioning, increasing vulnerability to and the impact of other diseases.

In South Africa, high TB/HIV co-infection rates are an important contributory factor to increased TB risk in farmworkers. Population TB risk is elevated an additional 25% by HIV [74,75]. In 2020, Africa was reported as the region with the highest cases of HIV/TB co-infection, with South Africa contributing to an astounding 50% of these [76]. HIV was directly responsible for 55% (4.8 million) of the TB cases and 69% (1.4 million) of TB deaths [77].

As described elsewhere [30], HIV infection alters the general immune system as well as specifically attacking *M. tuberculosis*-specific T cells, rendering individuals highly susceptible to developing active TB. Conversely, *M. tuberculosis* infection has a negative impact on the immune system’s response to HIV, increasing the likelihood of HIV infection and accelerating the progression from HIV infection to AIDS [78]. HIV and TB infections have also been found to impact the morbidity and mortality of other diseases, including COVID-19. A cohort study of the South African active hospital surveillance system for COVID-19 hospital admissions found that the odds of COVID-19 in-hospital mortality were higher in people with current or past TB infections (AOR 1.48, 95% CI 1.32–1.67) and for those with HIV (1.34, 1.27–1.43) [79].

HIV-related inflammation, toxicity of treatments, and increasing longevity of people living with HIV increases vulnerability to chronic communicable diseases, including MetS [80]. Modern HIV treatments are known to result in an increase in body weight [81,82,83]. Excess body weight has consequential spillover effects on other metabolic outcomes, including increased risks for diabetes and hypertension [84,85]. Poorly managed HIV, including detectable and/or fluctuating viral load levels, has been shown to be associated with higher comorbid rates with hypertension, diabetes, and cardiovascular diseases [80,85,86].

Alcohol use is a risk factor for TB, HIV, and MetS. A meta-analysis determined that alcohol use is associated with a 35% higher risk of TB compared to no alcohol use, which increased to 56% in high-TB burden countries [87]. Furthermore, TB risk rises as ethanol intake increases, with ethanol intake of more than 60 g per day associated with a 68% higher risk of tuberculosis compared to no alcohol use [87]. Problem drinking increases HIV exposure through increased likelihood of sexual violence, and reduced engagement with condom use [88,89]. Finally, a meta-analysis of prospective studies found that problem drinkers had a 84% higher risk of MetS than non-drinkers [90].

HIV, TB, and MetS can all be aggravated by high levels of pesticide exposure. Aside from acute poisonings that can occur, persistent pesticide exposure can disrupt immune system function by inhibiting lymphocytes and monocyte proliferation, affecting the production of cytokines and immunoglobulins, altering the cell’s phagocytic activity, and inducing apoptosis, thereby increasing infectivity and worsening progression of HIV and TB [91,92]. Long-term exposure to pesticides has the potential to induce energy metabolic disorders by disturbing the physical process of energy absorption in the intestine and energy storage in the liver, adipose tissue, and skeletal muscle, as well as energy regulation by the pancreas and immune cells [93]. These pesticide-induced disruptions ultimately cause abnormal levels of blood glucose and lipids, which in turn induce the development of related metabolic diseases, including overweight, underweight, and diabetes [94]. Pesticide exposure can also increase risk for chronic respiratory illnesses, malnutrition, depression, and problem drinking [95,96,97,98,99].

The second aspect of a syndemic—structural factors creating unsafe occupational conditions that drive disease—is also evident on farms in the Cape Winelands. Many argue that occupational health hazards in the Western Cape are related partly to the legacy of institutionalized distribution of alcohol by means of the “dop” (tot) system, a form of remuneration practiced during the colonial and apartheid eras whereby farmworkers were part-paid with low-quality wine supplied throughout the day [100,101,102,103]. This practice was a means to control and maintain farmworkers as a cheap source of labor and was not restricted to workers involved in the wine industry. The “dop” system and its legacy of inexpensive and readily available alcohol reflects the wider dispersion of a culture of alcohol consumption in rural communities in the region today [68].

Current labor policies further contribute to compounding disease burden among wine farmworkers. As noted previously, the ESTA, aiming to protect workers from housing evictions, has resulted in worse housing conditions, or no housing, as employers are unable to evict terminated employees, resulting in less housing availability. Employers are not required to provide housing, and under SD13, are not allowed to deduct housing costs from wages. SD13 also requires employers to provide benefits, such as paid sick leave, as described previously. To reduce this cost, employers offer only limited-term contracts that are not subject to the same benefit and labor regulation requirements. Furthermore, union formation for wine farmworkers has been blocked and there is limited labor policy compliance oversight (see “Efforts to address occupational syndemics in Cape Winelands”). In addition, the centralization of healthcare services limits access to rural farm dwellers with limited access to transportation.

Singer termed these conditions of employment a form of “occupational violence” [37]. Over time this produces chronic occupational stress experienced both physiologically and emotionally as constant fatigue, anxiety, feelings of hopelessness, and the sense of being a disposable commodity [41]. Chronic stress induces enduring exposure to stress-related hormones (e.g., corticotrophin-releasing hormone, cortisol, catecholamines, and thyroid hormone). Prolonged exposure to these chemical messengers, which places the body in a constant state of alertness, weakens the immune system, affects muscle and cognitive function, and promotes mental health issues [104], increasing the risk for multiple interacting health conditions and the development of occupational syndemics.

## 6. Efforts to Address Occupational Syndemics in Cape Winelands

Efforts to improve conditions on farms in the Cape Winelands have been made by various private actors, including farmers’ associations, industry bodies, and retailers [39]. In 2001, Agri Wes-Cape, the largest farmers’ association in the province and the provincial affiliate of Agri SA, the largest agricultural organization in South Africa, adopted a comprehensive Code of Conduct for its members. In 2002, the wine industry created the Wine Industry Ethical Trade Association (WIETA), a multi-stakeholder initiative that audits members. In 2008, the fruit industry began an ethical trade program. Some international retailers have imposed their own audit requirements and supported other programs within their supply chains.

During the COVID-19 pandemic, the WIETA collaborated with government institutions and industry bodies to obtain information and guidance on how to mitigate the pandemic. In response to the pandemic, in 2022, 24 farmworkers were trained as community health workers, a Western Cape Government Health initiative in collaboration with non-profit and agricultural industry partners [105]. The Aurum Institute in Malmesbury, Western Cape, implemented the USAID-funded TB Local Organizations Network (LON) project in 2022 to support organizations to implement locally generated solutions to improve TB diagnosis, treatment, and prevention services [106].

These initiatives have had varying degrees of reach and impact, but have so far failed to substantially alter conditions across all farms in the Western Cape, both because the state’s lack of enforcement of farmworkers’ rights [107,108] and recent shifts in global health and development funding. In 2013, the ratio of labor inspectors to workers in the Western Cape was 1:16,090, exceeding the International Labor Organization’s recommended ratio of 1:10,000. At the time, Inspection and Enforcement Services in the Department of Labor had only 56 inspectors in the Western Cape, of which just 6 were Occupational Health Safety inspectors. Given that these 56 inspectors had to inspect all workplaces in the province, they only managed to conduct 23% of inspections in the Agriculture, Forestry and Fishing sector [109]. In addition, an agreement between the Department of Labor, AgriSA, and other parties requires that labor inspectors give farmers prior notice of inspections, undermining inspectors’ capacity to identify violations [39].

The COVID-19 pandemic further challenged efforts to improve occupational conditions. With an influx of COVID-19 patients admitted to medical facilities, South Africa observed a 50% reduction in the number of TB tests performed and in the collection of HIV and TB medication during pandemic years [110,111]. As facilities and resources were reallocated to treat COVID-19 patients, other patients with major diseases, like HIV or TB, were at risk of not being treated or of developing complications [112,113]. Similar disruptions in HIV testing, positive HIV tests, and initiation of antiretroviral therapy were reported as an impact of COVID-19 [114]. Models project that this will dramatically increase incidence of both HIV and TB for years to come [115].

In January 2025, the USAID issued blanket stop-orders to pause the implementation of USAID-funded activities, and in February terminated all USAID-funded programs in South Africa. In South Africa, PEPFAR and USAID make up 14% of the national TB budget. The reduction in funding has impacted linkage to TB care, prevention, screening, testing, treatment and follow-up, community-based services, and TB-HIV integration, threatening progress in reducing TB incidence and mortality, and undermining the significant progress made in South Africa in addressing both HIV and TB over the past decade [116].

## 7. Conclusions

The syndemics lens affords insights into the sociobiological dynamics of health as codetermined by interacting biological and social forces. This examination of wine farmworkers in South Africa indicates that these forces include physically demanding and dangerous working conditions, labor policies which support substandard and unsanitary living environments, labor migration, precarious working contracts, minimal benefits and low pay, and a centralized healthcare system that limits access to robust healthcare. In addition, farmworkers in the Western Cape region are still impacted by the historical practice of the “dop” system, which created a culture of problem alcohol use. These conditions of occupational violence increase the susceptibility of wine farmworkers to high levels of chronic stress, which increases susceptibility to communicable and non-communicable diseases that present as an occupational syndemic. The syndemics framework offers an important tool to understand the impact of national and global social and structural relationships and their impact on worker health and can be used to inform public health and clinical responses and comprehensive labor reforms.
